# Supplementary material for: Tracking of Internal Granular Progenitors Responding to Valproic Acid in the Cerebellar Cortex of Infant Ferrets
Source: Cells. 2024 Feb 7;13(4):308. doi: 10.3390/cells13040308 (PMC10886983; doi:10.3390/cells13040308)
Supplement: Supplementary file 1 [file cells-13-00308-s001.zip › Figure S1.pdf]

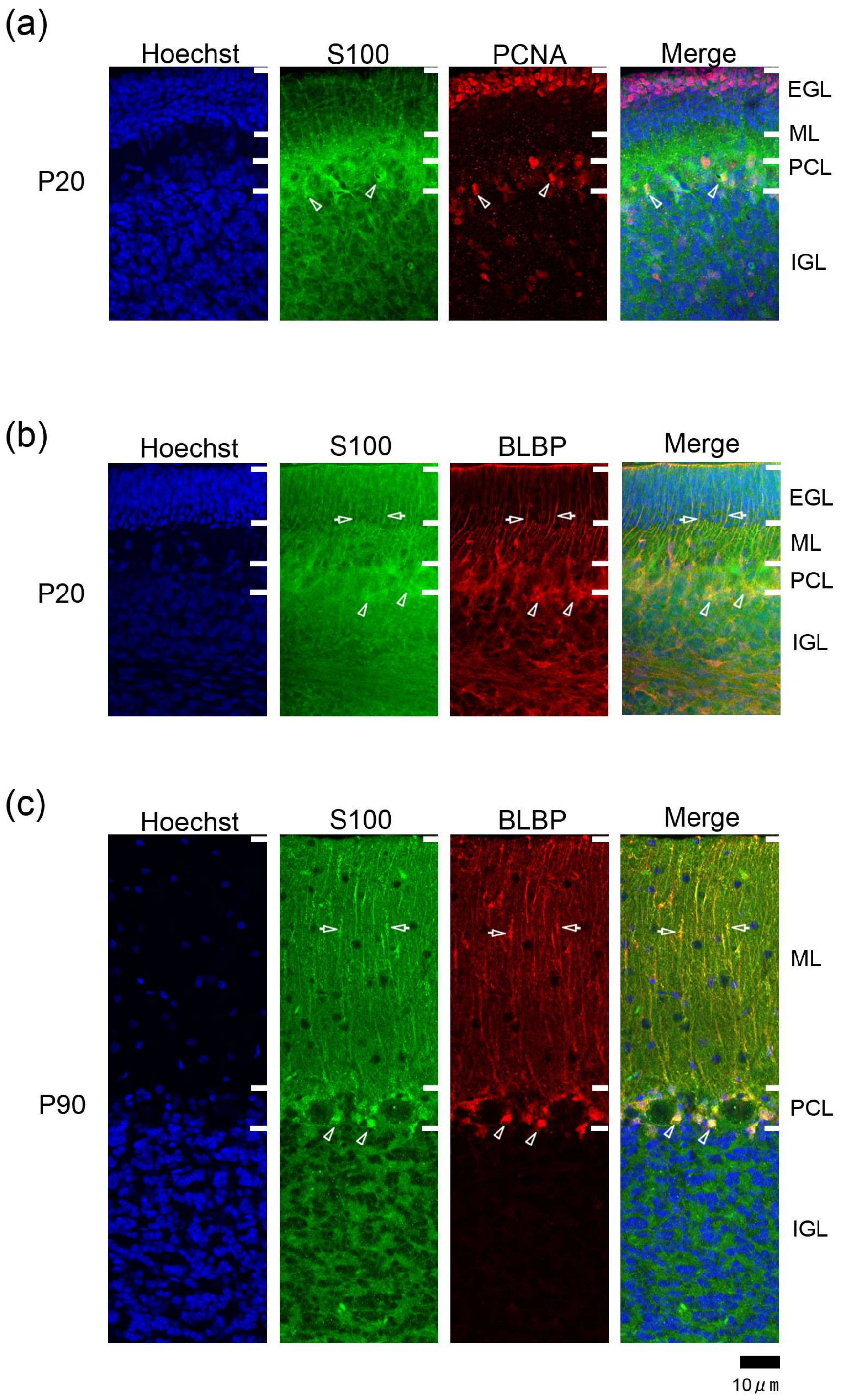

Figure S1. Immunofluorescence staining of S100, PCNA, and BLBP in the cerebellar cortex of PD 20 ferrets. (a) Double staining of S100 and PCNA in the cerebellar cortex of PD 20 ferrets; some cell bodies of S100-positive Bergmann glia show PCNA immunopositivity (arrowheads). (b) Double staining of S100 and BLBP in cerebellar cortex of PD 20 ferrets; some cell bodies of S100-positive Bergmann glia show BLBP immunopositivity (arrowheads). Some Bergmann glial processes are double positive for S100 and BLBP (arrows). (c) Double staining of S100 and BLBP in the cerebellar cortex of young adult ferrets. Most S100-positive Bergmann glia cell bodies are BLBP immunopositive (arrowheads). Most Bergmann glial processes are double positive for S100 and BLBP (arrows). EGL, external granular layer; IGL, internal granular layer; ML, molecular layer; PCL, Purkinje cell layer.
