# Supplementary material for: Tracking of Internal Granular Progenitors Responding to Valproic Acid in the Cerebellar Cortex of Infant Ferrets
Source: Cells. 2024 Feb 7;13(4):308. doi: 10.3390/cells13040308 (PMC10886983; doi:10.3390/cells13040308)
Supplement: Supplementary file 1 [file cells-13-00308-s001.zip › Table S1.pdf]

Table S1. Primary antibodies used in this study.

Antigens	Hosts	Concentration used	Cat#	RRDD	Source
BrdU	Rat monoclonal	1:500	ab6326	AB_305426	Abcam
BrdU	Sheep polyclonal	1:500	ab1893	AB_302659	Abcam
Calbindin-D28k	Rabbit polyclonal	1:500	CB38	AB_10000340	Swant
Parvalbumin	Mouse monoclonal	1:500	PV235	AB_10000393	Swant
NeuN	Rabbit polyclonal	1:500	ABN78	AB_10807945	Millipore
Pax6	Mouse monoclonal	1:500	ab78545	AB_1566562	Abcam
PCNA	Mouse monoclonal	1:500	MAB424	AB_95106	Millipore
S100	Rabbit polyclonal	1:500	942001	AB_572261	ImmunoStar
BLBP	Rabbit polyclonal	1:500	ab32423	AB_880078	Abcam
