# Supplementary material for: Tracking of Internal Granular Progenitors Responding to Valproic Acid in the Cerebellar Cortex of Infant Ferrets
Source: Cells. 2024 Feb 7;13(4):308. doi: 10.3390/cells13040308 (PMC10886983; doi:10.3390/cells13040308)
Supplement: Supplementary file 1 [file cells-13-00308-s001.zip › Table S2.pdf]

Table S2. Secondary antibodies used in this study.

Secondary antibody	Hosts	Concentration used	Cat#	Source
Alexa Fluor 555 anti-mouse	Donkey	1:500	A31570	Thermo Fisher Scientific
Alexa Fluor 647 anti-rabbit	Donkey	1:500	A31571	Thermo Fisher Scientific
Alexa Fluor 350 Anti-rat	Goat	1:500	A21093	Thermo Fisher Scientific
Alexa Fluor 350 Anti-sheep	Donkey	1:500	A21097	Abcam
