# Supplementary material for: Tracking of Internal Granular Progenitors Responding to Valproic Acid in the Cerebellar Cortex of Infant Ferrets
Source: Cells. 2024 Feb 7;13(4):308. doi: 10.3390/cells13040308 (PMC10886983; doi:10.3390/cells13040308)
Supplement: Supplementary file 1 [file cells-13-00308-s001.zip › Table S3.pdf]

Table S3. Cerebellar volume of VPA-exposed and control ferrets on postnatal day 20.

	n	Volume (mm^3)
Control	4	177.2 ± 23.5
VPA	4	170.8 ± 23.6

Mean ± Standard error. The cerebellar volume was estimated using MR images that had been obtained from brain samples of ferrets exposed to VPA with the same administration schedule as in a previous study (Sawada et al. PLoS One, 2021)[20].
